# Patient Characteristics and Treatment Outcomes of Nasopharyngeal Carcinoma in Nonendemic Regions

**DOI:** 10.1001/jamanetworkopen.2025.1895

**Published:** 2025-03-26

**Authors:** Mohammad Bilal Alsavaf, Matthew Marquardt, Moataz D. Abouammo, Menglin Xu, Ahmed Elguindy, John Grecula, Sujith Baliga, David Konieczkowski, Emile Gogineni, Priyanka Bhateja, James W. Rocco, Matthew O. Old, Dukagjin M. Blakaj, Ricardo L. Carrau, Kyle K. VanKoevering, Marcelo Bonomi

**Affiliations:** 1 Department of Otolaryngology-Head and Neck Surgery, The Ohio State University Wexner Medical Center, Columbus; 2 The Ohio State University College of Medicine, Columbus; 3 Department of Internal Medicine, Division of Medical Oncology, The Ohio State University Wexner Medical Center, Columbus; 4 Division of Radiation Oncology, The Ohio State University Wexner Medical Center, Columbus

## Abstract

**Importance:**

Nasopharyngeal carcinoma (NPC) presents unique challenges in nonendemic regions, with varying patient characteristics and outcomes compared with endemic populations.

**Objective:**

To fill gaps in the current understanding of NPC by focusing on a US population, comparing patient characteristics and treatment outcomes with endemic populations, and identifying key factors to inform management and follow-up protocols in Western health care settings.

**Design, Setting, and Participants:**

This retrospective cohort study included patients with NPC treated at a single large US tertiary academic medical center from 2000 to 2023. The study analyzed patient demographics, tumor characteristics, treatment modalities, and survival outcomes. Data were analyzed from January to July 2024.

**Main Outcomes and Measures:**

Overall survival (OS), progression-free survival (PFS), and recurrence-free survival, stratified by patient characteristics, tumor types, Epstein-Barr virus (EBV) status, and p16 expression.

**Results:**

The sample included 159 adult patients with NPC (median [range] age, 53.5 [18-90] years; 117 [73.6%] male), with 23 African American patients (15.3%), 21 Asian patients (14.0%), and 106 White patients (70.7%). World Health Organization type III tumors predominated (88 patients [68.8%]), followed by type II (25 patients [19.5%]) and type I (15 patients [11.7%]). EBV positivity rates varied significantly by race (Asian: 13 patients [81.3%]; African American: 17 patients [63.0%]; White: 40 patients [47.0%]; *P* = .03) and WHO type (type III: 50 patients [72.5%]; type II: 10 patients [48.0%]; type I: 0 patients; *P* < .001). p16 status, a proxy for human papillomavirus status, did not vary by race but did vary by histopathologies (type III: 12 patients [28.5%]; type II: 12 patients [63.0%]; type I: 3 patients [43.0%]; *P* = .04). On Kaplan-Meier curves, stratifying p16 by EBV status eliminated its assumed association with OS. Multivariate analysis revealed that increasing age (hazard ratio [HR] per 1-year increase, 1.03 [95% CI, 1.00-1.05]; *P* = .04) and former smoking status (HR, 2.29 [95% CI, 1.03-5.10]; *P* = .04) were associated with inferior OS, while WHO type III tumors were associated with better OS compared with type I (HR, 0.38 [95% CI, 0.17-0.87]; *P* = .02). Male sex was associated with worse PFS (HR, 5.35 [95% CI, 1.23-23.30]; *P* = .03). For recurrence-free survival, former smokers (HR, 25.24 [95% CI, 2.56-249.23]; *P* = .006), current smokers (HR, 44.97 [95% CI, 2.27-892.10]; *P* = .01), and patients with advanced stages (IVa/b) (HR, 261.34 [95% CI, 3.96-17 258.06]; *P* = .009) had significantly increased risk.

**Conclusions and Relevance:**

This cohort study contributes to the evolving body of knowledge on NPC in nonendemic regions, finding a shift toward WHO type III tumors and underscoring the association of EBV status with survival outcomes, while highlighting the lack of association between human papillomavirus status and outcomes. Smoking history, advanced stage at diagnosis, male sex, and increasing age emerged as adverse factors. Notably, WHO type I tumors demonstrated particularly poor outcomes, highlighting the need for more intensive follow-up in this subgroup.

## Key Points

**Question:**

Are racial, histological, and other characteristics associated with outcomes among patients with nasopharyngeal carcinoma (NPC) in a nonendemic region, and have presentation patterns evolved compared with those in endemic regions?

**Findings:**

In this cohort study that included 159 patients with NPC, World Health Organization (WHO) type III tumors predominated across all racial groups, with Epstein-Barr virus positivity varying significantly by race. Age, smoking history, male gender, Epstein-Barr virus status, and WHO type I disease were associated with worse survival outcomes.

**Meaning:**

These findings suggest that NPC presentation patterns in nonendemic regions have shifted toward WHO type III predominance, with Epstein-Barr virus status and smoking history remaining key factors associated with survival outcomes.

## Teaser

This cohort study describes patient characteristics and treatment outcomes among patients with nasopharyngeal carcinoma in the US, a nonendemic region.

## Introduction

Nasopharyngeal carcinoma (NPC) is a rare cancer that exhibits a distinct geographic distribution focused around East and Southeast Asia and North Africa, where incidence ranges from 4 to 25 cases per 100 000 individuals.^1^ In 2022, approximately 83% of worldwide NPCs occurred in Asia, with the incidence of NPC in endemic regions 50 to 100 times greater than elsewhere.^2,3^ The incidence of NPC in first-generation immigrants from endemic areas remains high, while second-generation individuals show reduced but still elevated rates.^1,4^

The World Health Organization (WHO) classifies NPC into 3 subtypes based on cellular morphology: type I (keratinizing), found predominately in nonendemic areas, and types II (nonkeratinizing) and III (undifferentiated) are found predominately in endemic regions.^5^ Type II is the most common, representing more than 95% of NPCs in endemic regions; however, type I accounts for more than 75% of NPCs in the US. While there is most likely a multifactorial etiology to NPC tumorigenesis, viral infection is likely a key driver. In endemic regions, Epstein-Barr virus (EBV) infection has long been associated with type II NPC, while human papillomavirus (HPV) has emerged as another potential factor, particularly in type I NPC, with very few individuals being coinfected with EBV.^1,5,6,7,8,9,10^

A significant challenge in NPC management is that more than 50% of patients present with advanced-stage disease—defined as stage III or IV—at the time of diagnosis, which complicates treatment and negatively affects survival.^1,5^ Concurrent chemoradiotherapy (CCRT) has become the cornerstone for treating locally advanced NPC, with induction chemotherapy (ICT) or adjuvant chemotherapy (ACT) increasingly added, given the superior outcomes.^1,5,11,12^ Screening for EBV, which can facilitate earlier detection, has also helped improve outcomes.^13,14,15^ The incidence of NPC has progressively declined, with some endemic regions seeing 3% to 5% reductions annually over 10- to 20-year periods.^16,17,18^ While overall outcomes have improved through earlier detection and new treatment regimens, the 5-year survival for advanced-stage NPC remains at approximately 50%, suggesting a need for new prognostic indicators that could facilitate screening and treatment.^19,20^

Most clinical studies on NPC originate from Asia; however, more than 2000 NPCs are estimated to occur in the US annually.^3^ Through this retrospective study, we present our experience treating a diverse cohort of patients at a single, large US academic medical center, aiming to fill the gaps in the current understanding of NPC in nonendemic regions. We identify potentially important factors and determine overall survival (OS) and progression-free survival (PFS) in a diverse cohort, which can be used to inform more effective and individualized treatment strategies for patients in nonendemic regions.

## Methods

This cohort study was approved by the institutional review board of The Ohio State University and Wexner Medical Center. Informed consent requirement was waived due to the study’s observational retrospective design. This study follows the Strengthening the Reporting of Observational Studies in Epidemiology (STROBE) reporting guideline for observational cohort studies.

### Study Design and Patient Selection

We conducted a retrospective review of patient electronic medical records at The Ohio State University Wexner Medical Center between 2000 and 2023. Patients were identified through *International Statistical Classification of Diseases and Related Health Problems, Tenth Revision* (*ICD-10*) diagnosis codes for NPC and confirmed via their final histopathological analysis. From the patients who were initially identified, those who had missing final pathology, nonprimary, recurrent, and non-NPC histology tumors were excluded.

### Data Collection

We extracted demographic, clinical, pathological, and treatment data from electronic medical records. Variables collected included age at diagnosis, sex, race and ethnicity, smoking history, alcohol consumption, tumor histology (WHO classification), tumor stage (American Joint Committee on Cancer, eighth edition), EBV status, p16 status, treatment modalities, and survival outcomes. Race and ethnicity categories were self-reported by patients during hospital registration as African American, Asian, or White. Race and ethnicity data were collected to assess potential demographic disparities in outcomes, given the known variation in disease prevalence across populations.

All cases underwent review by experienced head and neck pathologists and adhered to WHO classification criteria: type I (keratinizing squamous cell carcinoma), type II (nonkeratinizing carcinoma), and type III (undifferentiated carcinoma). EBV positivity was determined through quantitative polymerase chain reaction measurement of plasma EBV DNA levels. Positive PCR results were often confirmed, and negative results were always verified, using EBV encoding region in-situ hybridization (EBER-ISH) performed on tumor tissue specimens. p16 expression was used as a surrogate marker for HPV and determined via immunohistochemistry staining, with positivity defined as nuclear and cytoplasmic staining in at least 70% of tumor cells.

### Treatment Protocols, Follow-Up, and End Points

Radiation and chemotherapy treatment protocols are outlined in the (eMethods 1 in Supplement 1). Patients were followed from the date of pathological diagnosis until death or last clinical contact. Follow-up assessments occurred every 3 months for the first 2 years, then biannually thereafter. The primary outcome was OS, defined as the time from treatment completion to death from any cause. Secondary outcomes included PFS, recurrence-free survival (RFS), and metastasis-free survival (MFS), all measured from treatment completion to event occurrence or last follow-up.

### Statistical Analysis

Detailed description of the statistical tests used and how analyzes were conducted can be found in (eMethods 2 in Supplement 1). In brief, 2-sample *t* tests were conducted for univariate associations between outcomes and independent variables of interest, analysis of variance tests were used for continuous variables, and Fisher exact tests were carried out for dichotomous or categorical variables. Pearson correlation coefficient (*r*) was used to assess the strength and direction of the linear association between continuous parameters. Cox proportional hazards regression models were used to evaluate the associations of patient characteristics and selected variables with OS, PFS, RFS, and MFS. Kaplan-Meier estimators were calculated to estimate the survival functions for time-to-event data from which survival curves were plotted, and log-rank tests were used to test the differences between groups of interest. *P* values were 2-sided, and statistical significance was set at *P* < .05. We used JASP version 0.18.3 (The JASP Team), Jamovi version 2.5.3 (jamovi project), and R statistical software version 4.3.0 (R Project for Statistical Computing) for statistical analyses. Data were analyzed from January 2024 to July 2024.

## Results

### Baseline Patient Characteristics

A total of 224 patients were initially identified, with 159 included in the final cohort. For the study cohort, median (range) follow-up time was 39 (0-273) months. The median (range) age at diagnosis was 53.5 (18-90) years, with a male predominance (117 [73.6%] male). Among 150 patients with race and ethnicity data, there were 23 African American patients (15.3%), 21 Asian patients (14.0%), and 106 White patients (70.7%). More than one-third of patients (57 patients [35.8%]) were current smokers, nearly one-third (47 patients [29.6%]) were former smokers, and the remaining 55 patients (34.6%) were never smokers. Patient age varied significantly by smoking status (mean [SD] age: nonsmokers, 48.66 [16.11] years; former smokers, 59.51 [12.28] years; current smokers, 53.28 [14.46] years; *P* = .001). Current smokers had a mean (SD) of 36.0 (25.6) pack-years, and former smokers had 29.1 (33.2) pack-years, with mean (SD) durations of smoking of 31.4 (14.2) years and 22.7 (15.1) years, respectively. Alcohol consumption was reported by 61 patients (38.4%), while 23 patients (14.5%) reported marijuana use (Table 1).

**Table 1.** Baseline Patient Characteristics and Treatment Details

Characteristic	Patients, No. (%)				*P* value
	Overall	Treatment (N = 155)			
		ICT + CCRT	CCRT	CCRT + ACT	
Sample size	159 (100)	48 (31.0)	59 (38.1)	48 (31.0)	NA
Age at diagnosis, mean (SD), y	53.5 (15.0)	51.6 (16.4)	54.6 (16.7)	53 (11.1)	.57
Sex					
Male	117 (73.6)	36 (75.0)	41 (69.5)	38 (79.0)	.52
Female	42 (26.4)	12 (25.0)	18 (30.5)	10 (21.0)	
Race					
African American	23 (15.3)	15 (31.3)	11 (18.6)	6 (12.5)	.23
Asian	21 (14)	5 (10.4)	9 (15.3)	7 (14.6)	
White	106 (70.7)	28 (58.3)	39 (66.1)	35 (72.9)	
Smoking					
Current	57 (35.8)	15 (31.3)	23 (39.0)	18 (37.5)	.88
Former	47 (29.6)	15 (31.3)	15 (25.4)	15 (31.3)	
Never	55 (34.6)	18 (37.5)	21 (35.6)	15 (31.3)	
Alcohol					
Yes	61 (38.4)	23 (47.9)	22 (37.3)	14 (29.2)	.16
No	98 (61.6)	25 (52.1)	37 (62.7)	34 (70.8)	
Marijuana					
Yes	23 (14.5)	9 (18.8)	5 (8.5)	8 (16.7)	.27
No	136 (85.5)	39 (81.3)	54 (91.5)	40 (83.3)	
WHO histological type					
I	15 (11.7)	4 (12.1)	7 (14.6)	3 (7.0)	.73
II	25 (19.5)	7 (21.2)	10 (20.8)	7 (16.3)	
III	88 (68.8)	22 (66.7)	31 (64.6)	33 (76.7)	
Missing, No.	31	15	11	5	
AJCC stage					
I and II	27 (18.0)	9 (18.8)	10 (19.2)	7 (14.9)	.41
III	53 (35.3)	15 (31.25)	22 (42.3)	16 (34.0)	
IVa and IVb	70 (46.7)	24 (50)	20 (38.5)	24 (51.1)	
Missing, No.	9	0	7	1	
EBV status					
Positive	70 (56.0)	28 (59.6)	23 (51.1)	19 (57.6)	.005
Negative	55 (44.0)	19 (40.4)	22 (48.9)	14 (42.4)	
Missing, No.	30	1	14	15	
p16 status					
Positive	38 (42.2)	12 (36.4)	16 (48.5)	8 (38.1)	.12
Negative	52 (57.8)	21 (63.6)	17 (51.5)	13 (61.9)	
Missing, No.	69	15	26	27	

Abbreviations: ACT, adjuvant chemotherapy; AJCC, American Joint Committee on Cancer (eighth edition); CCRT, concurrent chemoradiotherapy; EBV, Epstein-Barr virus; ICT, induction chemotherapy; WHO, World Health Organization.

The most common histological subtype was WHO type III (88 patients [68.8%]), followed by type II (25 patients [19.5%]) and type I (15 patients [11.7%]). Histological type data were missing for 31 patients. According to the American Joint Committee on Cancer staging system, 70 patients (46.7%) presented with locally advanced stage IVa or IVb, 53 patients (35.3%) presented with stage III, and 27 patients (18.0%) presented with early stage I or II disease. EBV status was positive for 70 patients (56.0%) and negative for 55 patients (44.0%). p16 expression was positive for 38 patients (42.2%) and negative for 52 patients (57.8%). For treatment modalities, 48 patients (31.0%) received ICT followed by CCRT, 59 patients (38.0%) received CCRT alone, and 48 patients (31.0%) received CCRT followed by ACT (Table 1). Tumor recurrence was detected primarily through imaging (26 patients [74.3%]), followed by clinical symptoms (9 patients [25.7%]), and routine physician examination (3 patients [8.6%]), with all cases confirmed histologically via biopsy. Sensitivity analyses revealed no significant differences in baseline characteristics between patients with complete vs missing EBV and p16 data.

### Associations of Smoking Status, EBV, and p16 With WHO Type and Staging

EBV varied significantly across WHO histological types (50 patients [72.5%] with type III disease; 10 patients [48.0%] with type II; 0 patients with type I; *P* < .001), while p16 positivity showed an inverse association with histological types (12 patients [28.5%] with type III; 12 patients [63.0%] with type II; 3 patients [43.0%] with type I; *P* = .04). However, neither EBV nor p16 status was significantly associated with tumor staging. Patient age significantly differed across WHO types, with mean (SD) ages of 64.8 (13.18) years, 56.24 (12.08) years, and 50.26 (16.35) years for types I, II, and III, respectively (*P* = .002) (Table 2).

**Table 2.** Associations of Smoking Status, EBV, and p16 With WHO Type and AJCC Staging

Characteristic	WHO type				AJCC staging			
	Patients, No. (%)			*P* value	Patients, No. (%)			*P* value
	I	II	III		I and II	III	IVa and IVb	
Age, mean (SD), y	65 (13)	56 (12)	50 (16)	.002	55 (15)	53 (15)	53 (14)	.86
Smoking								
Current	4 (26.7)	8 (32.0)	29 (33.0)	.03	6 (22.2)	15 (28.3)	32 (45.7)	.16
Former	9 (60.0)	11 (44.0)	21 (24.0)		9 (33.3)	17 (32.1)	18 (25.7)	
Never	2 (13.3)	6 (24.0)	38 (43.0)		12 (44.5)	21 (39.6)	20 (28.6)	
EBV								
Positive	0	10 (48.0)	50 (72.5)	<.001	17 (71.0)	27 (55.0)	26 (51.0)	.27
Negative	14 (100)	11 (52.0)	19 (27.5)		7 (29.0)	22 (45.0)	25 (49.0)	
p16								
Positive	3 (43.0)	12 (63.0)	12 (28.5)	.04	5 (69.0)	15 (44.0)	18 (46.0)	.63
Negative	4 (57.0)	7 (37.0)	30 (71.5)		11 (31.0)	19 (56.0)	21 (54.0)	

Abbreviations: AJCC, American Joint Committee on Cancer (eighth edition); EBV, Epstein-Barr virus; WHO, World Health Organization.

### Racial Differences in EBV Status, p16 Status, WHO Types, and Tumor Staging

Significant racial differences were observed in EBV positivity, with the highest frequency in Asian patients (13 patients [81.3%]) followed by African American patients (17 patients [63.0%]) and then White patients (40 patients [47.0%]; *P* = .03). Most tumors were WHO type III, and WHO type did not vary significantly by race. There were no statistically significant differences in early-stage (I or II) disease (African American: 4 patients [12.5%]; Asian: 7 patients [37.0%]; White: 16 patients [16.2%]; *P* = .13). Underlying tumor characteristics, including p16 status, histological type, and overall staging, did not vary significantly by race (Table 3).

**Table 3.** Racial Differences in EBV Status, p16 Status, WHO Type, and Tumor Staging

Characteristic	Patients by race and ethnicity, No. (%)			χ^2^	*P* value
	African American	Asian	White		
EBV status					
Negative	10 (37.0)	3 (18.8)	45 (53.0)	7.298	.03
Positive	17 (63.0)	13 (81.3)	40 (47.0)		
p16 status					
Negative	13 (65.0)	8 (88.9)	31 (50.8)	5.209	.08
Positive	7 (35.0)	1 (11.1)	30 (49.2)		
WHO histological type					
I	3 (12.0)	1 (5.3)	11 (13.1)	4.639	.36
II	5 (20.0)	1 (5.3)	19 (22.6)		
III	17 (68.0)	17 (89.4)	54 (64.3)		
AJCC stage					
I and II	4 (12.5)	7 (37.0)	16 (16.2)	7.622	.13
III	14 (43.8)	7 (37.0)	32 (32.3)		
IVa and IVb	14 (43.8)	5 (26.0)	51 (51.5)		

Abbreviations: AJCC, American Joint Committee on Cancer (eighth edition); EBV, Epstein-Barr virus; WHO, World Health Organization.

### Survival Analysis

#### Univariate Analysis

On univariate analysis, female sex was associated with better outcomes for recurrence, metastasis, PFS, and OS (eTable 1 in Supplement 1) . WHO type (III vs I) was associated with reduced recurrence and improved OS. Early-stage disease (I/II vs IVa/b) was associated with better OS (eTable 1 in Supplement 1).

Factors associated with worse OS included older age at diagnosis, current smoking (compared with former and never smokers), and EBV negativity (eTable 1 in Supplement 1). Additionally, for ever-smokers, each additional year of smoking was associated with increasing the risk of overall death by 4% (hazard ratio [HR], 1.04 [95% CI, 1.02-1.06]; *P* < .001), while each additional pack per year increased the risk of overall death by 26% (HR, 1.26 [95% CI, 1.03-1.54]; *P* = .02).

#### Multivariate Analysis

**Figure 1.**
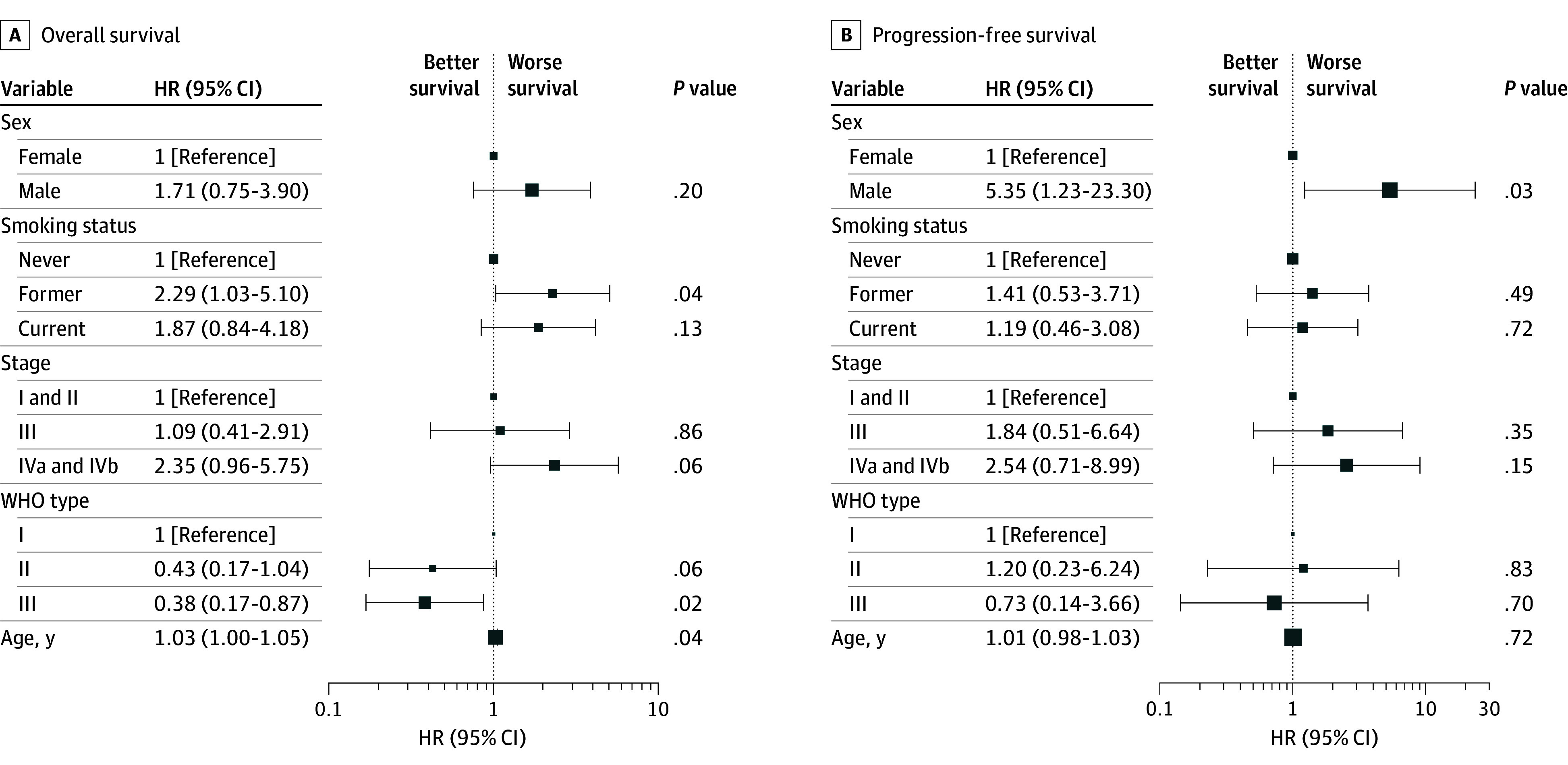
Multivariate Cox Regression Analysis of Factors Associated With Overall and Progression-Free Survival HR indicates hazard ratio; WHO, World Health Organization. Size of square indicates sample size.

**Figure 2.**
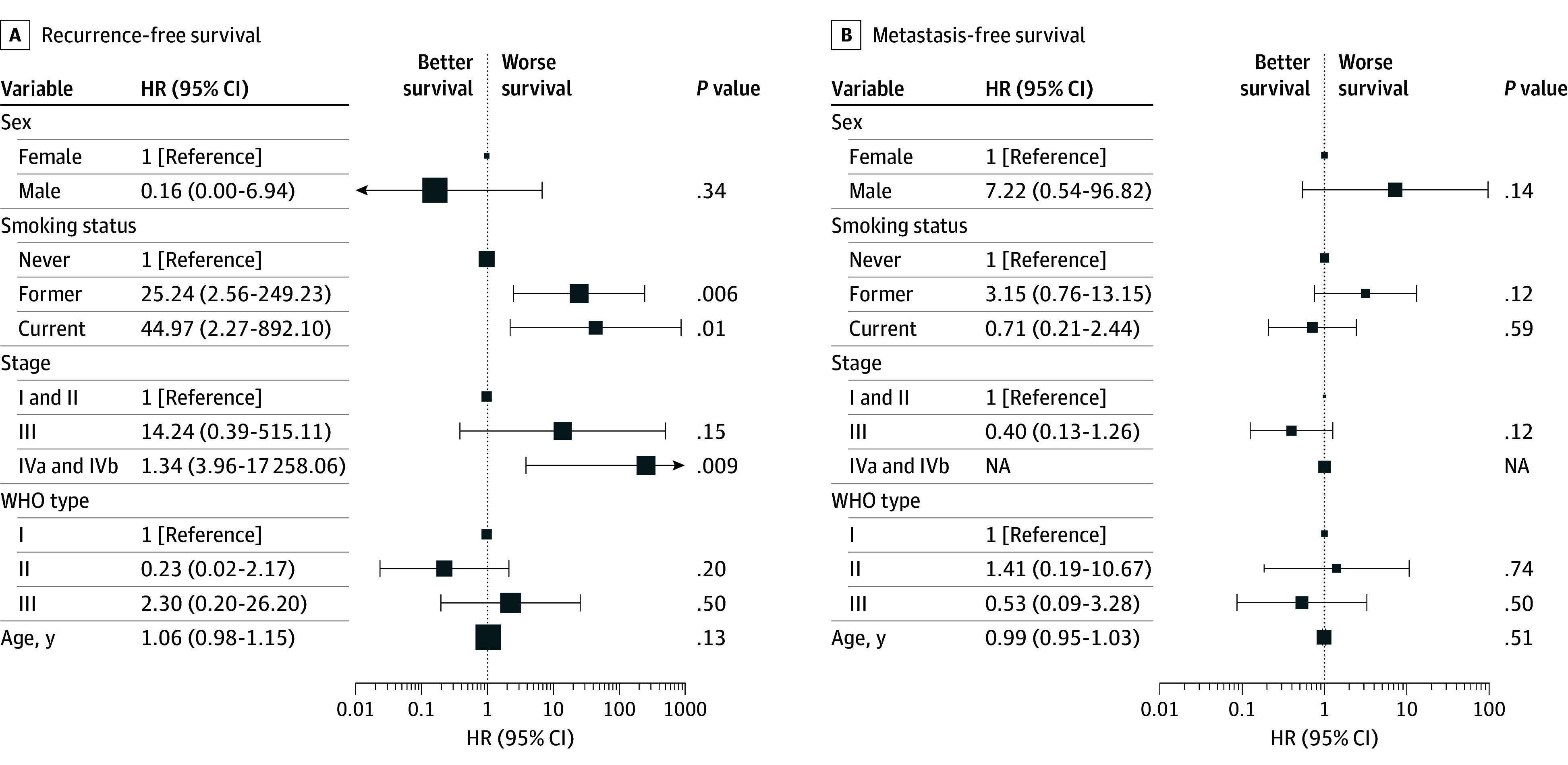
Multivariate Cox Regression Analysis of Factors Associated With Recurrence-Free and Metastasis-Free Survival HR indicates hazard ratio; NA, not applicable; WHO, World Health Organization. Size of square indicates sample size.

In multivariate Cox regression analysis, significant factors associated with OS included age (HR per 1-year increase, 1.03 [95% CI, 1.00-1.05]; *P* = .04) and former smoking status compared with nonsmokers (HR, 2.29 [95% CI, 1.03-5.10]; *P* = .04). WHO type III disease was associated with better outcomes compared with type I (HR, 0.38 [95% CI, 0.17-0.87]; *P* = .02). For PFS, male sex was associated with worse outcomes (HR: 5.35 [95% CI, 1.23-23.30]; *P* = .03). Recurrence-free survival was significantly worse for patients with advanced-stage disease (IVa/b) (HR, 261.34 [95% CI, 3.96-17 258.06]; *P* = .009), former smokers (HR, 25.24 [95% CI, 2.56-249.23]; *P* = .006), and current smokers (HR, 44.97 [95% CI, 2.27-892.10]; *P* = .01). No statistically significant associations were found for MFS in the multivariate analysis (Figure 1 and Figure 2). p16 positivity was not associated with OS, PFS, RFS, or MFS. The 1-, 3-, 5-, and 10-year OS and PFS rates overall and across different factors were analyzed and summarized in eTable 2 and eTable 3 in Supplement 1.

### Kaplan-Meier Survival Analyses

Overall survival differed significantly by sex (HR, 1.89 [95% CI, 1.10-3.25]; *P* = .02), smoking status (HR, 2.31 [95% CI, 1.48-3.60]; *P* = .001), EBV status (HR, 0.48 [95% CI, 0.30-0.77]; *P* = .003), WHO type (HR, 3.15 [95% CI, 2.11-4.70]; *P* < .001), and tumor stage (HR, 2.83 [95% CI, 1.98-4.05]; *P* < .001). Female patients, never-smokers, patients with positive EBV status, patients with type I and II tumors, and patients with earlier-stage disease had superior survival probabilities. Race was not significantly associated with OS. At 12 months, patients type I disease showed 26.7% (95% CI, 11.5%-61.7%) OS; type II, 76.0% (95% CI, 61.0%-94.7%) with 61.0% risk reduction (*P* = .02); and type III, 89.7% (95% CI, 83.5%-96.3%) with 77% risk reduction (*P* <.001) compared with type I.

For PFS, sex emerged as a significant factor (HR, 1.92 [95% CI, 1.23-3.01]; *P* = .004) with female patients showing better outcomes. Interestingly, EBV status was not significantly associated with PFS. There were also no differences between OS, PFS, RFS, or MFS when comparing treatment modalities (ICT with CCRT vs CCRT alone vs CCRT with AC; ICT with CCRT and CCRT with ACT vs CCRT) (eFigures 1-13 in Supplement 1).

## Discussion

This cohort study is the first large-scale, single-center study of NPC in a US population examining evolving patient characteristics, racial differences, and treatment outcomes, to our knowledge. National databased studies of NPC are limited by potential data entry errors and frequently lack crucial information on EBV status, p16 expression, and lifestyle factors, necessitating more comprehensive single-center analyses.^21,22^ Through the identification and analysis of potential risk factors, we offer valuable insights to inform NPC management and follow-up protocols in Western health care settings.

Two national studies conducted in the US in 1998 and 2013 highlighted a predominance of type I NPC with a slight shift toward type III, potentially attributed to an increase in EBV-related NPC.^21,22,23^ However, we found a marked shift toward type III NPC in both Asian and White populations. In our cohort, EBV-positive tumors were still more common in Asian patients compared with African American and White patients. A 2019 study by Chen et al^1^ reported data from Asian populations indicating a decreasing incidence rate of NPC, despite the EBV positivity ratio remaining the same.^1^ This trend, coupled with changes in presentation patterns in migrant populations,^4^ underscores the multifactorial etiology of NPC, suggesting a complex interplay between environmental factors and genetic predisposition in its development. It is believed that high-risk populations exhibit specific human leukocyte antigen haplotypes that impair anti-EBV immune responses in nasopharyngeal epithelium.^24^ Also, nonendemic populations exhibit lower EBV antigen antibody titers, suggesting regional host-virus interaction variations.^25^ Notably, a stepwise reduction in NPC incidence has been observed as successive generations of high-risk populations have migrated and intermarried with low-risk indigenous groups.^26^ Other studies emphasize NPC’s multifactorial nature by demonstrating that disease pathogenesis is influenced by smoking, chronic rhinitis, faucitis, chronic bronchitis, tuberculosis, and hepatitis.^26^

NPC associated with p16 overexpression and HPV oncogenesis has been of increasing interest in recent years. The increasing incidence of oropharyngeal cancer in North America and Western Europe has been attributed to oncogenic HPV/p16 status and has been shown to significantly influence treatment response and outcomes.^27^ While Singhi et al^28^ hypothesized that HPV-related NPC might be an extension of oropharyngeal disease, Robinson et al^29^ found that NPC is a distinct entity without contiguous spread. Some studies have reported that p16 expression is associated with improved outcomes in NPC, potentially due to enhanced radiosensitivity.^30,31^ Our investigation found varying p16 prevalence across racial groups, with the lowest rates among Asian patients, and WHO subtypes. Importantly, when stratifying p16 status by EBV status, our data suggest that EBV, rather than p16, is the primary driver in NPC prognosis and that EBV-driven tumors might also demonstrate p16 overexpression.^32^

The role of p16 in cancer progression is more nuanced than initially believed, with overexpression observed in malignant neoplasms beyond HPV-related tumors. Elevated levels of wild-type and mutant p16 are paradoxically associated with poor outcomes in several cancers, challenging our understanding of its tumor-suppressive function.^33^ While p16 overexpression is a common surrogate marker for HPV infection in oropharynx squamous cell carcinomas, it can occur independently of HPV status, influenced by diverse molecular mechanisms involving cyclin D1, gankyrin, p34^SEI-1^, cell division cycle 6, and nuclear factor κ-B.^33^ In some tumors, p16 alteration overexpression may attempt to restore functionality, possibly triggered by cellular stress or oncogenic factors. However, its antiproliferative effects might be counteracted by alternative molecular events, highlighting the complex interplay between tumor suppression and promotion.^34^ The underlying causes of HPV/p16 overexpression in cancers remain complex and poorly understood, emphasizing the need for further research to unravel its multifaceted role in cancer biology.

Treatment advancements for locoregionally advanced NPC have significantly improved OS and quality of life, starting with the landmark 0099 study that demonstrated enhanced OS with the addition of ACT.^35,36,37,38,39^ Subsequent trials and meta-analyses, including NPC-9901 and NPC-9902, highlighted ACT’s role in enhancing PFS, while ICT was shown to provide superior distant control in locally advanced NPC.^12,40,41,42^ A 2024 meta-analyses reported that taxane- and platinum-based ICT followed by CCRT potentially offers the best efficacy and cost-effectiveness.^43^ However, previous studies were primarily conducted on EBV-positive populations. Our study, examining a mixed population of patients with and without EBV in the US, found no significant differences in OS, PFS, RFS, or MFS among treatment modalities analyzed.

Univariate analysis revealed significantly better OS, PFS, RFS, and MFS in females compared with males. However, multivariate Cox analysis showed significance only for PFS. These findings aligned with previous studies from endemic areas that reported better prognoses and more adverse treatment events in females.^44,45^ Notably, this female advantage was observed only during premenopausal and menopausal periods, disappearing in the postmenopausal phase, which might suggest a protective effect of estrogen.^45,46^ We also found advanced age to be significantly associated with worse OS, which corroborates the rapid increase in disease-related mortality after age 45 in males and 55 in females, as reported by Li et al.^44^ This age-related decline in outcomes could be attributed to reduced tolerance for high-dose treatments or to altered pharmacodynamics and pharmacokinetics in older patients.^47^ Interestingly, while a study by Wu et al^48^ found that younger patients tend to present with more advanced disease at diagnosis, our study did not replicate this finding.

Smoking significantly increases the risk of developing NPC, with past or current smokers facing a 32% higher risk, escalating to 67% for those who smoked at least three-quarters of a pack per day.^49^ Moreover, smoking not only influences NPC development but also adversely affects treatment outcomes.^50^ Sun et al^51^ reported significantly worse survival and recurrence rates in current smokers, with both current and former smokers at higher risk of metastasis.^51^ Our study corroborates these findings, demonstrating significantly higher local recurrence risk in both current and former smokers. Interestingly, we observed significantly worse OS only in former smokers, possibly due to their higher mean age compared with current smokers. Notably, survival time was significantly associated with years of smoking and pack-years. This finding may explain why some ever-smokers, despite the association with EBV activation, still exhibit better prognoses.^52^ The detrimental impact of smoking on prognosis can be attributed to reduced sensitivity to CRT, as nicotine diminishes the cytotoxic effects of DNA-damaging agents.^53,54^ Additionally, smoking exacerbates tissue hypoxemia, inducing the expression of several malignancy-related genes.^55,56^

A study leveraging the Surveillance, Epidemiology, and End Results (SEER) database comparing histological subtypes of NPC found worse prognosis for type I compared with types II and III^57^; however, the SEER database lacks crucial data on EBV status, p16 expression, lifestyle factors, and treatment details. Our findings align with this trend, with particularly poor outcomes for type I NPC, especially during the first 12 months after treatment, underscoring the need for more frequent follow-up in this subgroup. Notably, we observed no EBV positivity among type I cases, coupled with a significantly higher mean age at diagnosis. While p16 positivity in type I was intermediate between types II and III, its prognostic significance remains unclear. Previous studies have suggested that type I NPC in the US might be attributable to smoking.^58,59^ While we did observe higher rates of ever-smoking in type I NPC, our data do not necessarily support this hypothesis.

### Limitations

This study has some limitations. As a single-center, retrospective study, our findings may not be fully generalizable to all nonendemic populations. Because of the relatively small sample size compared with endemic regions and significant cohort heterogeneity, a multicenter study would be valuable to validate these findings. Additionally, the long study period (2000-2023) spans significant changes in treatment protocols, which may have influenced outcomes. Also, while p16 immunohistochemistry was used as a surrogate marker for HPV status, as in some previous studies, HPV DNA testing would provide more definitive evidence of viral infection status. Although sensitivity analyses showed the missing EBV and p16 data were generally robust, these gaps may still affect the validity of related results.

## Conclusions

To our knowledge, this cohort study is the first large-scale, single-institution retrospective analysis in the US to shed light on NPC and address the limitations of national databased studies. Our findings highlighted the shifting histological patterns in both White and Chinese populations. EBV remains the most significant factor associated with survival outcomes, though the assumption that p16 expression improves outcomes was not supported after EBV stratification. We identified several adverse factors, including advanced age, male sex, smoking history, and advanced stage at diagnosis. Notably, WHO type I tumors were associated with poor outcomes, especially within the first year after treatment, highlighting the need for closer follow-up in this subgroup.

## Notes

Supplement 1. **eMethods 1.** Treatment Protocols **eMethods 2.** Statistical Analysis **eFigure 1.** Kaplan-Meier Overall Survival Analysis by Race. **eFigure 2.** Kaplan-Meier Overall Survival Analysis by Sex **eFigure 3.** Kaplan-Meier Overall Survival Analysis by Smoking Status **eFigure 4.** Kaplan-Meier Overall Survival Analysis by EBV Status **eFigure 5.** Kaplan-Meier Overall Survival Analysis by EBV and p16 Status **eFigure 6.** Kaplan-Meier Overall Survival Analysis by WHO Type **eFigure 7.** Kaplan-Meier Overall Survival Analysis by Tumor Stage **eFigure 8.** Kaplan-Meier Overall Survival Analysis of the Entire Cohort **eFigure 9.** Kaplan-Meier Progression-Free Survival Analysis of the Entire Cohort **eFigure 10.** Kaplan-Meier Progression-Free Survival Analysis by Sex **eFigure 11.** Kaplan-Meier Progression-Free Survival Analysis by EBV Status **eFigure 12.** Kaplan-Meier Progression-Free Survival Analysis by WHO Type **eFigure 13.** Kaplan-Meier Progression-Free Survival Analysis by Treatment Modality **eTable 1.** Univariate Cox Regression Analysis of Prognostic Factors for Recurrence, Metastasis, Progression-Free Survival, and Overall Survival **eTable 2.** Overall Survival Rates at 1, 3, 5, and 10 Years Across Different Prognostic Factors **eTable 3.** Progression-Free Survival Rates at 1, 3, 5, and 10 Years Across Different Prognostic Factors

Supplement 2.
Data Sharing Statement
