# Supplementary material for: Patient Characteristics and Treatment Outcomes of Nasopharyngeal Carcinoma in Nonendemic Regions
Source: JAMA Netw Open. 2025 Mar 26;8(3):e251895. doi: 10.1001/jamanetworkopen.2025.1895 (PMC11947841; doi:10.1001/jamanetworkopen.2025.1895)
Supplement: Supplement 1. — eMethods 1. Treatment Protocols eMethods 2. Statistical Analysis eFigure 1. Kaplan-Meier Overall Survival Analysis by Race. eFigure 2. Kaplan-Meier Overall Survival Analysis by Sex eFigure 3. Kaplan-Meier Overall Survival Analysis by Smoking Status eFigure 4. Kaplan-Meier Overall Survival Analysis by EBV Status eFigure 5. Kaplan-Meier Overall Survival Analysis by EBV and p16 Status eFigure 6. Kaplan-Meier Overall Survival Analysis by WHO Type eFigure 7. Kaplan-Meier Overall Survival Analysis by Tumor Stage eFigure 8. Kaplan-Meier Overall Survival Analysis of the Entire Cohort eFigure 9. Kaplan-Meier Progression-Free Survival Analysis of the Entire Cohort eFigure 10. Kaplan-Meier Progression-Free Survival Analysis by Sex eFigure 11. Kaplan-Meier Progression-Free Survival Analysis by EBV Status eFigure 12. Kaplan-Meier Progression-Free Survival Analysis by WHO Type eFigure 13. Kaplan-Meier Progression-Free Survival Analysis by Treatment Modality eTable 1. Univariate Cox Regression Analysis of Prognostic Factors for Recurrence, Metastasis, Progression-Free Survival, and Overall Survival eTable 2. Overall Survival Rates at 1, 3, 5, and 10 Years Across Different Prognostic Factors eTable 3. Progression-Free Survival Rates at 1, 3, 5, and 10 Years Across Different Prognostic Factors [file jamanetwopen-e251895-s001.pdf]

Supplemental Online Content

Alsavaf MB, Marquardt M, Abouammo MD, et al. Patient characteristics and treatment outcomes of nasopharyngeal carcinoma in nonendemic regions. *JAMA Netw Open*. 2025;8(3):e251895. doi:10.1001/jamanetworkopen.2025.1895

eMethods 1. Treatment Protocols
eMethods 2. Statistical Analysis
eFigure 1. Kaplan-Meier Overall Survival Analysis by Race.
eFigure 2. Kaplan-Meier Overall Survival Analysis by Sex
eFigure 3. Kaplan-Meier Overall Survival Analysis by Smoking Status
eFigure 4. Kaplan-Meier Overall Survival Analysis by EBV Status
eFigure 5. Kaplan-Meier Overall Survival Analysis by EBV and p16 Status
eFigure 6. Kaplan-Meier Overall Survival Analysis by WHO Type
eFigure 7. Kaplan-Meier Overall Survival Analysis by Tumor Stage
eFigure 8. Kaplan-Meier Overall Survival Analysis of the Entire Cohort
eFigure 9. Kaplan-Meier Progression-Free Survival Analysis of the Entire Cohort
eFigure 10. Kaplan-Meier Progression-Free Survival Analysis by Sex
eFigure 11. Kaplan-Meier Progression-Free Survival Analysis by EBV Status
eFigure 12. Kaplan-Meier Progression-Free Survival Analysis by WHO Type
eFigure 13. Kaplan-Meier Progression-Free Survival Analysis by Treatment Modality
eTable 1. Univariate Cox Regression Analysis of Prognostic Factors for Recurrence, Metastasis, Progression-Free Survival, and Overall Survival
eTable 2. Overall Survival Rates at 1, 3, 5, and 10 Years Across Different Prognostic Factors
eTable 3. Progression-Free Survival Rates at 1, 3, 5, and 10 Years Across Different Prognostic Factors

This supplemental material has been provided by the authors to give readers additional information about their work.

eMethods 1. Treatment Protocols

A. Radiation Therapy Protocol

Patients received intensity-modulated radiation therapy (IMRT) or volumetric modulated arc therapy (VMAT) to the primary tumor site and cervical nodal levels. The radiation regimen consisted of daily fractions administered five days per week. Accurate delineation of the primary tumor GTV (GTVp) and pathological nodes (GTVn) was achieved using the planning CT with contrast, and/or MRI neck, or PET/CT. Clinical target volume (CTV) delineation was done following the guidelines of the Radiation Therapy Oncology Group (RTOG) 0225 Phase II Trial and the international consensus guidelines.[22,23] CTV-high included GTVp and GTVn plus 3-5 mm with the addition of whole nasopharynx. CTV-intermediate included CTV high plus another 3-5mm, with the addition of the vomer, ethmoid and Sphenoid sinuses, cavernous sinus, skull base foramina superiorly, and entire parapharyngeal space, posterior 5mm of maxillary sinuses and nasal cavity anteriorly, and the anterior 1/3 of the clivus posteriorly. CTV-low included elective negative nodes that are at risk of microscopic disease (bilateral lateral retropharyngeal nodes, II, III and Va in all cases, and ipsilateral level 1b if involved or positive level II, and levels IV and Vb ipsilaterally if there are any involved lymph nodes). A 3-5mm margin was added to the CTV volumes to generate planning target volumes. The prescribed radiation doses were delivered over 33-35 fractions as follows:

1. Primary Tumor and pathological (or PET AVID) Lymph Nodes (CTV-high): 66.96 -70 Gy.
2. Intermediate-Risk Regions (CTV-intermediate): 5940-60 Gy.
3. Low-Risk Regions (CTV-low): 54-54.12 Gy.

B. Chemotherapy Protocol

Chemotherapy protocols included induction, concurrent, and adjuvant regimens based on platinum agents. Induction chemotherapy consisted of two regimens administered in three-week cycles for a total of three cycles:

1. TPF regimen: Docetaxel (75 mg/m2, day 1), Cisplatin (75 mg/m2, day 1), and 5-Fluorouracil (3000 mg/m2, days 1-5)
2. GemCis regimen: Gemcitabine (1000 mg/m2, days 1 and 8) and Cisplatin (75 mg/m2, day 1)

The CCRT regimen primarily used weekly single-agent Carboplatin (AUC 2) or Cisplatin (weekly 40mg/m2 or triweekly 100 mg/m2). Less frequently, Carboplatin (AUC 1) was combined with Paclitaxel (30 mg/m2), starting on the first day of radiotherapy.

The adjuvant chemotherapy regimen mainly used triweekly Carboplatin (AUC5) or Cisplatin (75 mg/m2) plus 5-Fluorouracil (3000 mg/m2 days 1-5). In two cases, Docetaxel (60 mg/m2) was used.

g

© 2025 Alsavaf MB et al. *JAMA Network Open.*

Means and standard deviations in the sample were utilized when variables were approximately normally distributed, while medians were used for skewed continuous variables. For categorical variables, the number and percentage of patients in the sample are shown for each level of the variable. Two-sample t-tests were conducted for univariate associations between outcomes and independent variables of interest, ANOVA test was utilized for continuous variables, and Fisher's exact test was carried out for dichotomous or categorical variables. Pearson's correlation coefficient (r) was used to assess the strength and direction of the linear relationship between continuous parameters. Cox proportional hazards regression models were employed to evaluate the effects of patient characteristics and selected variables on OS, PFS, RFS, and MFS. Kaplan-Meier estimators were calculated to estimate the survival functions for time-to-event data from which survival curves were plotted and log-rank tests were used to test the differences between groups of interest. To investigate the impact of missing data in EBV and p16 on the validity of the findings, we performed a sensitivity analysis examining if there is significant difference in the background variables between the valid vs missing observations. JASP (version 0.18.3), Jamovi (version 2.5.3), and R statistical software (version 4.3.0) were used for statistical analyses.

eFigure 1.

© 2025 Alsavaf MB et al. *JAMA Network Open.*

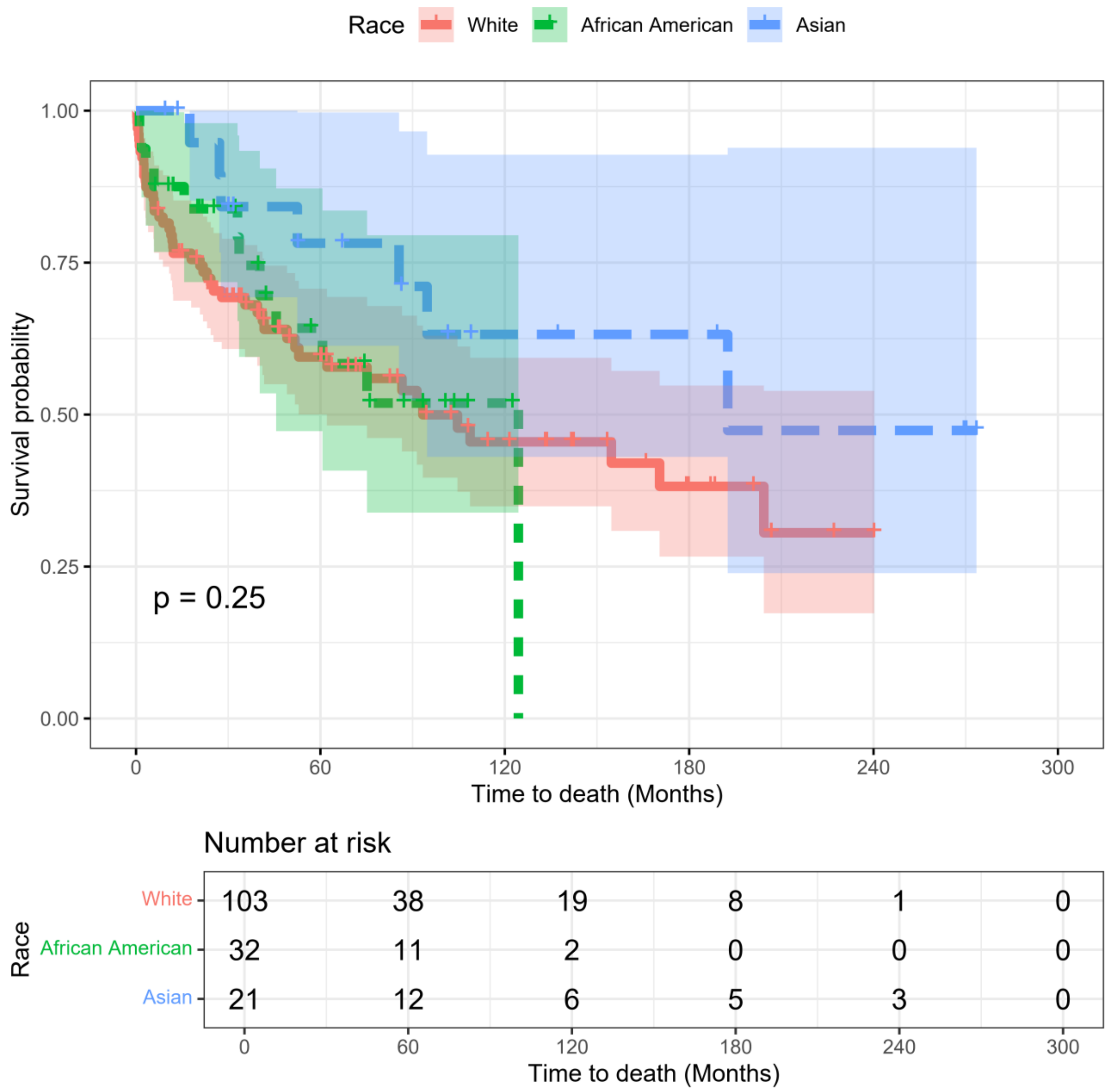

eFigure 2.

© 2025 Alsavaf MB et al. *JAMA Network Open*.

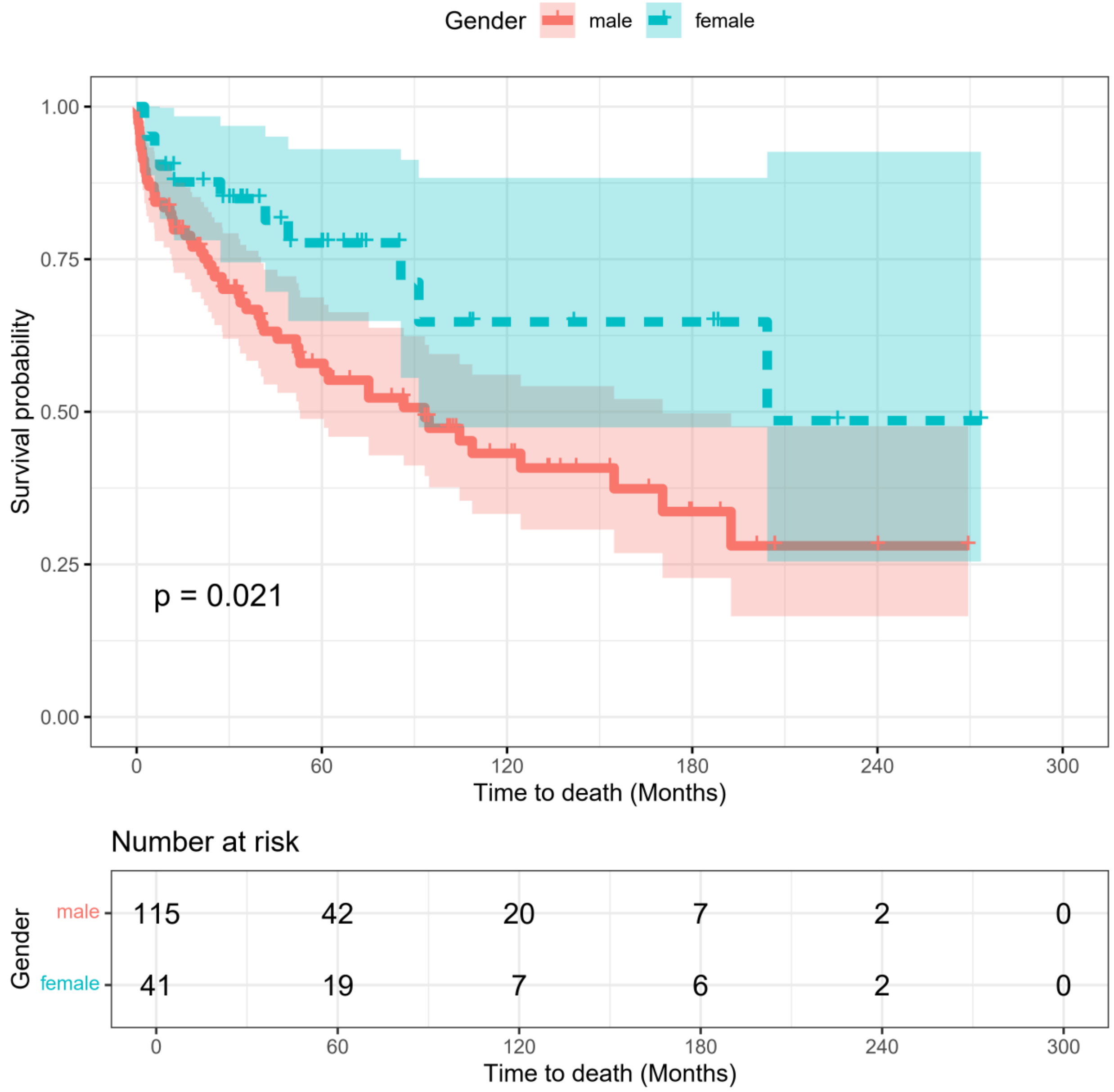

eFigure 3.

© 2025 Alsavaf MB et al. *JAMA Network Open*.

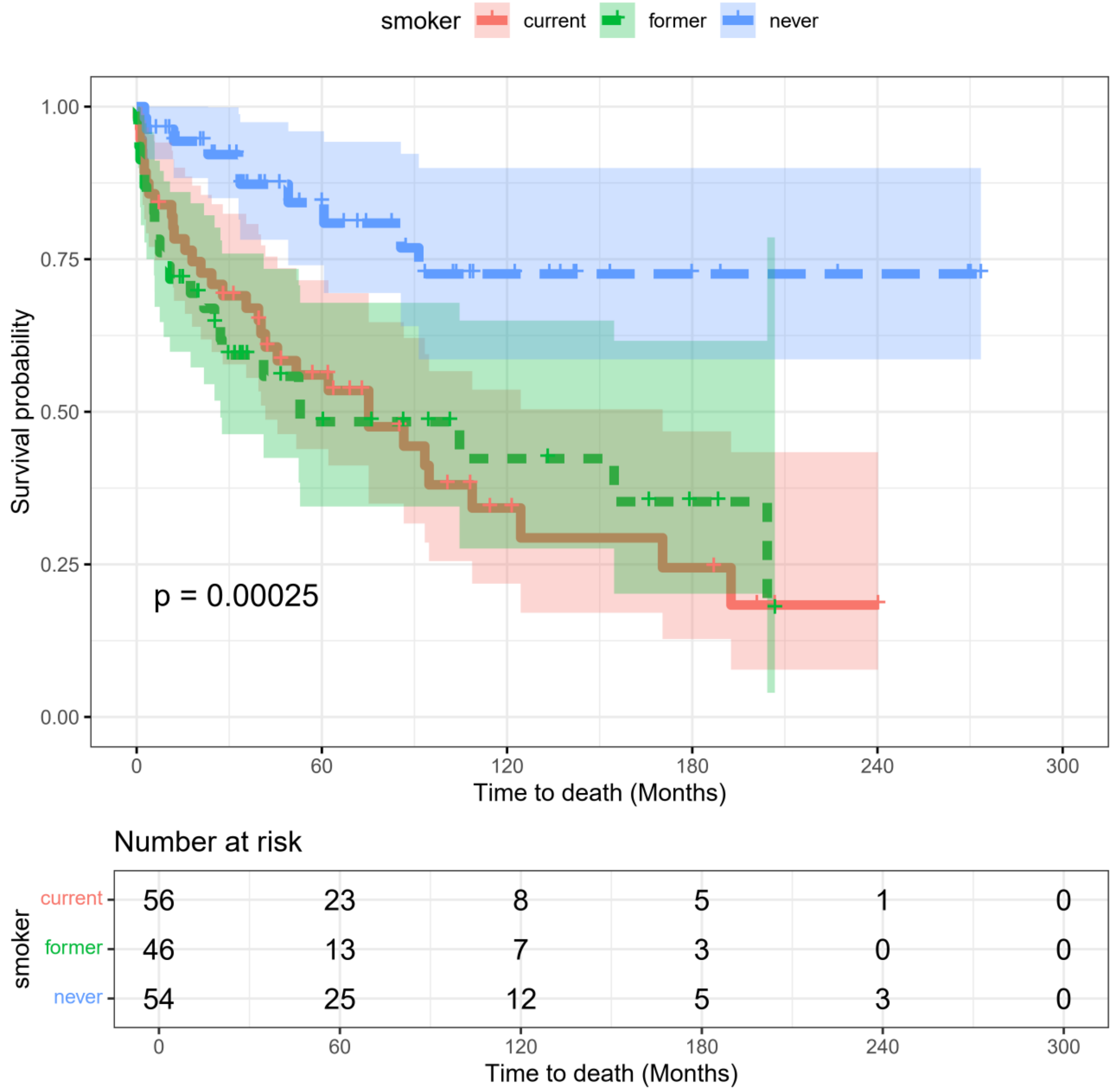

© 2025 Alsavaf MB et al. *JAMA Network Open*.

eFigure 4.

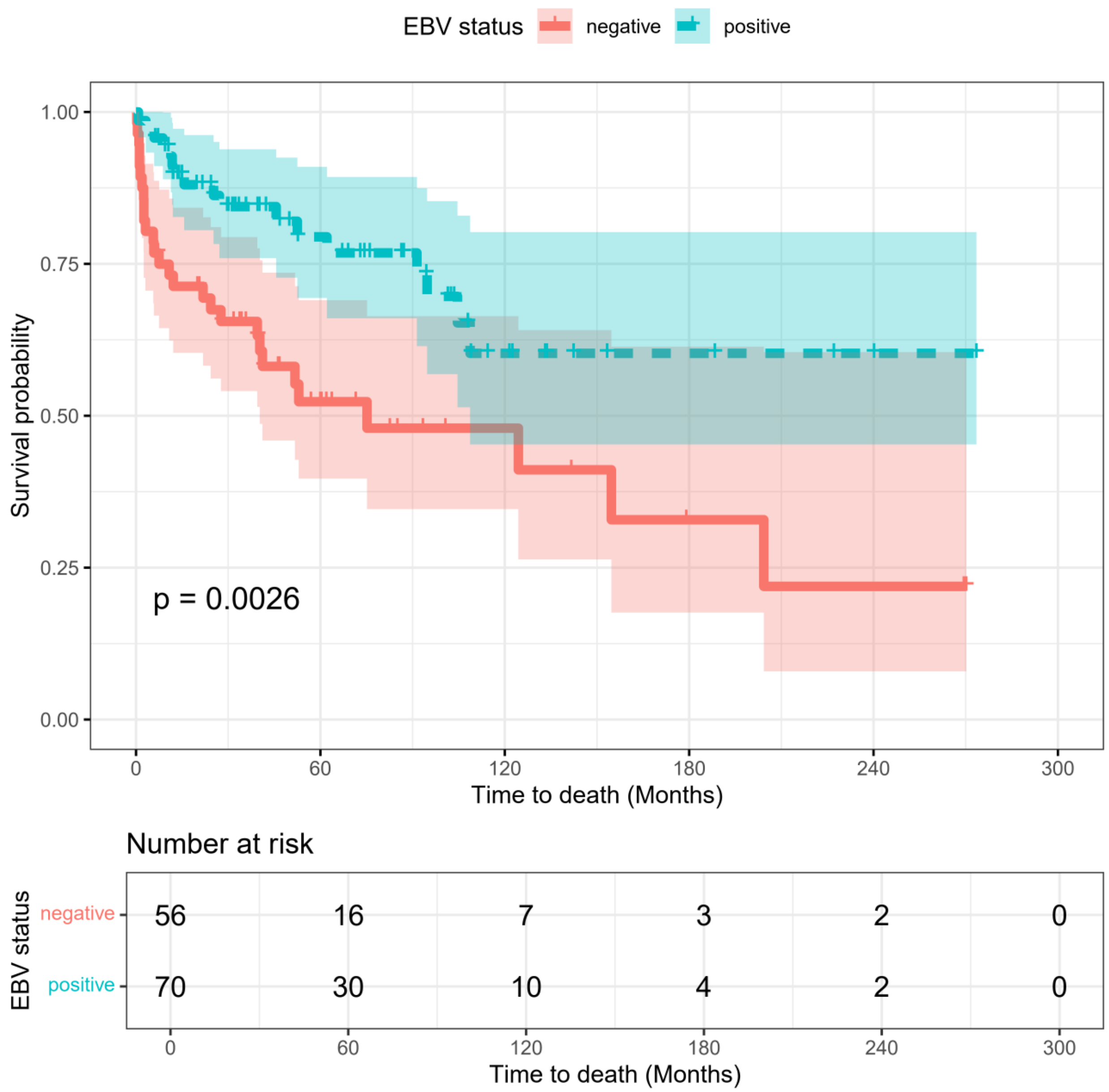

© 2025 Alsavaf MB et al. *JAMA Network Open*.

eFigure 5.

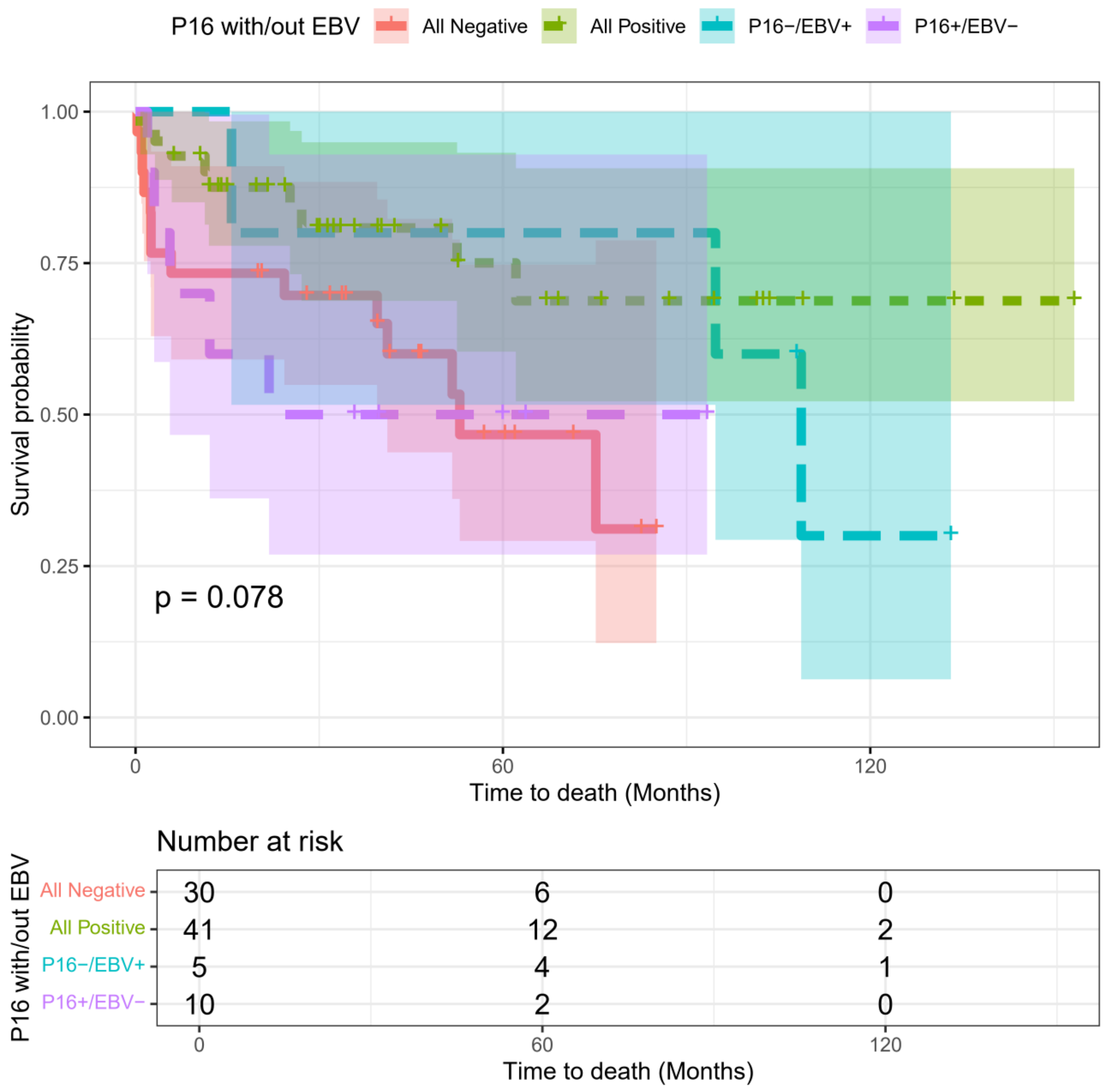

© 2025 Alsavaf MB et al. *JAMA Network Open*.

eFigure 6.

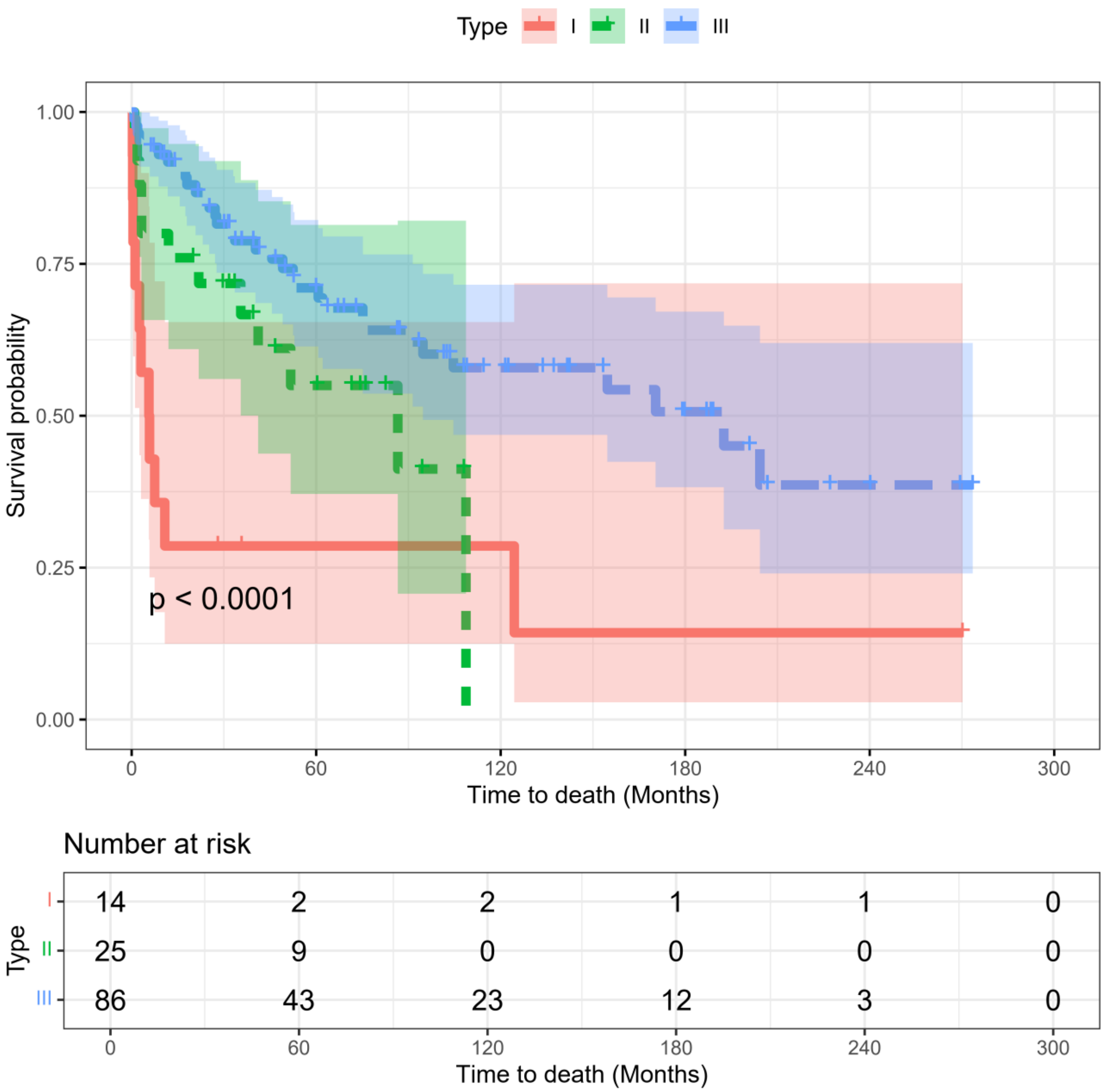

© 2025 Alsavaf MB et al. *JAMA Network Open*.

eFigure 7.

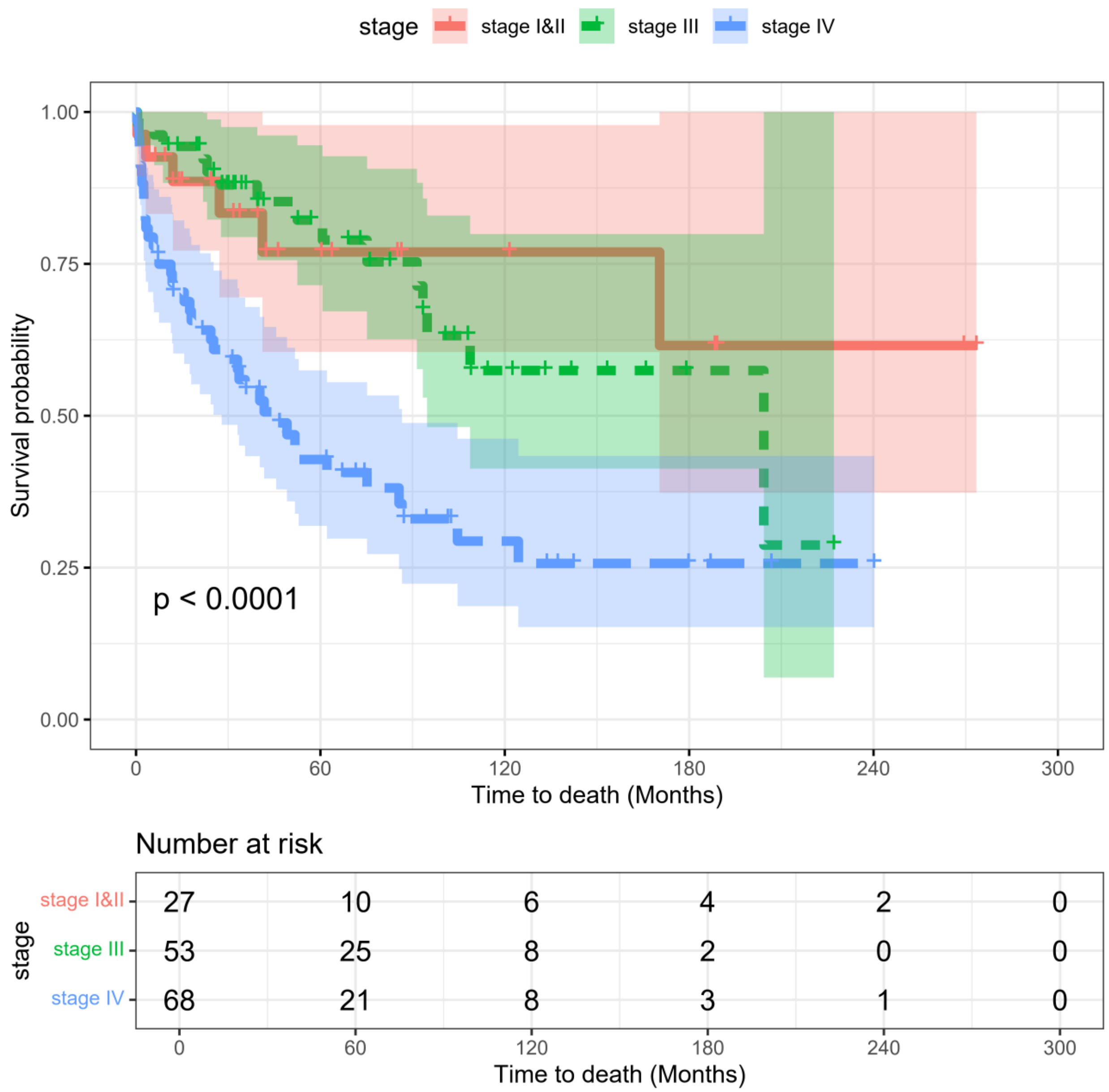

© 2025 Alsavaf MB et al. *JAMA Network Open*.

eFigure 8.

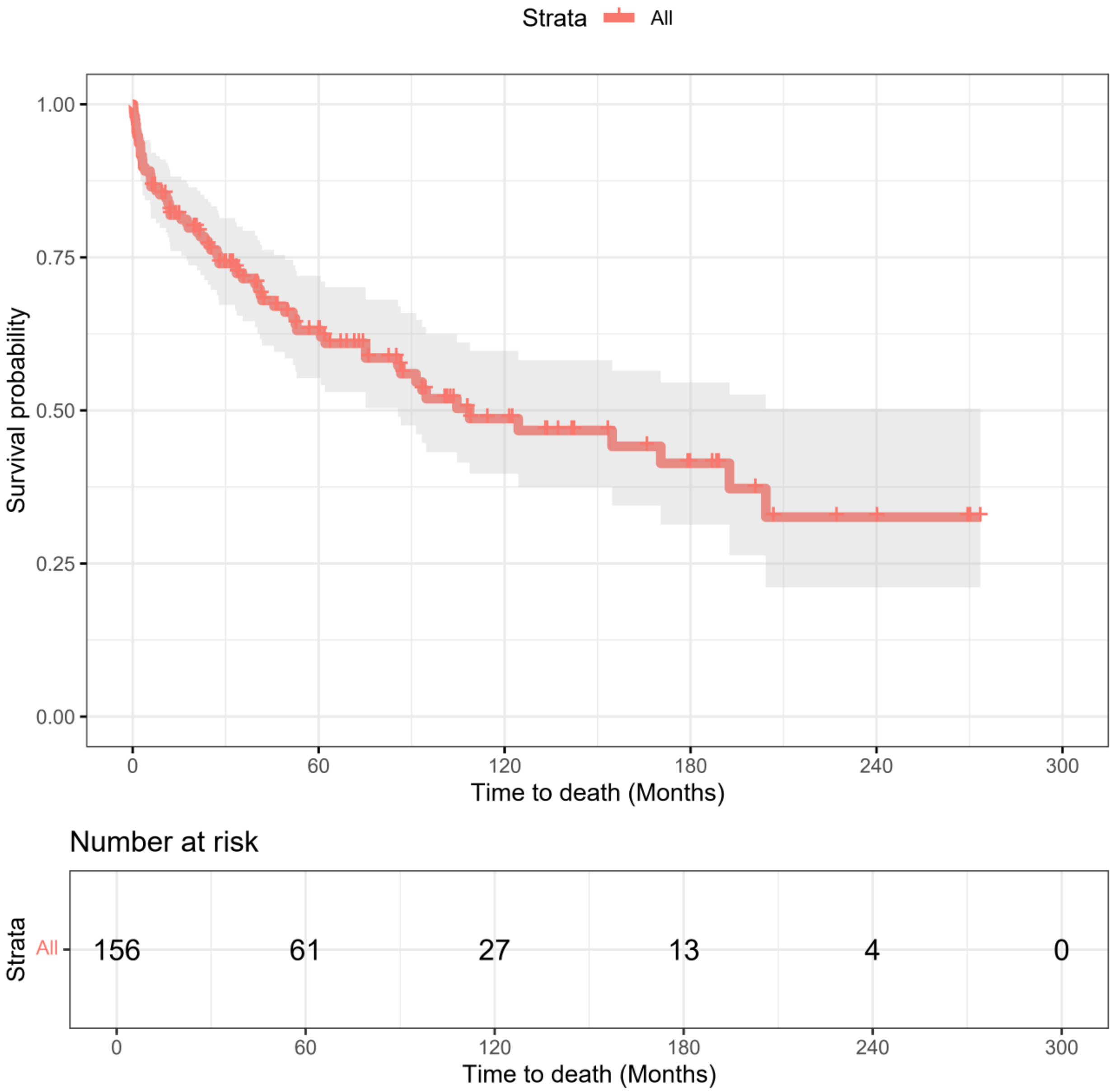

© 2025 Alsavaf MB et al. *JAMA Network Open*.

eFigure 9.

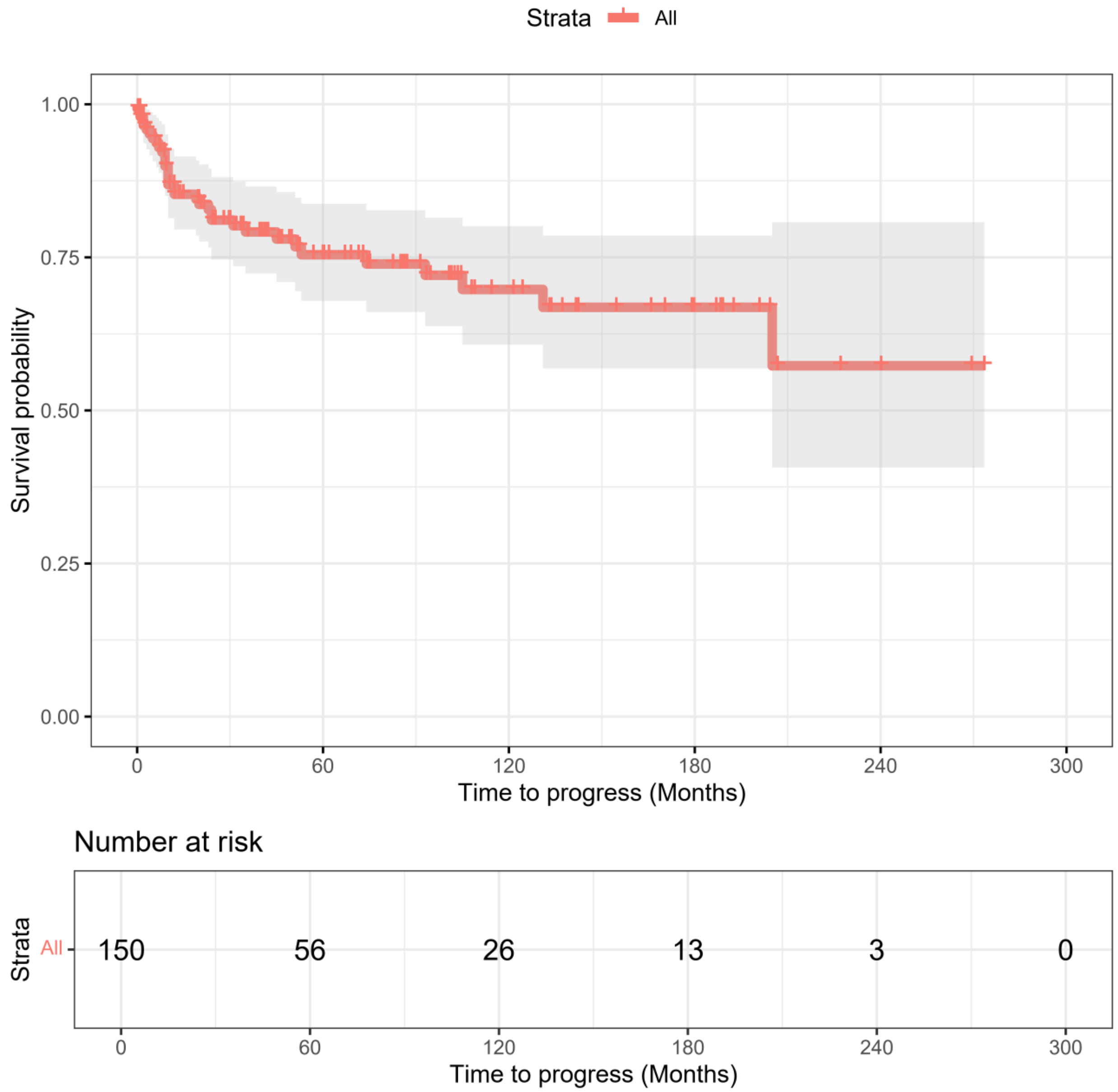

© 2025 Alsavaf MB et al. *JAMA Network Open*.

eFigure 10.

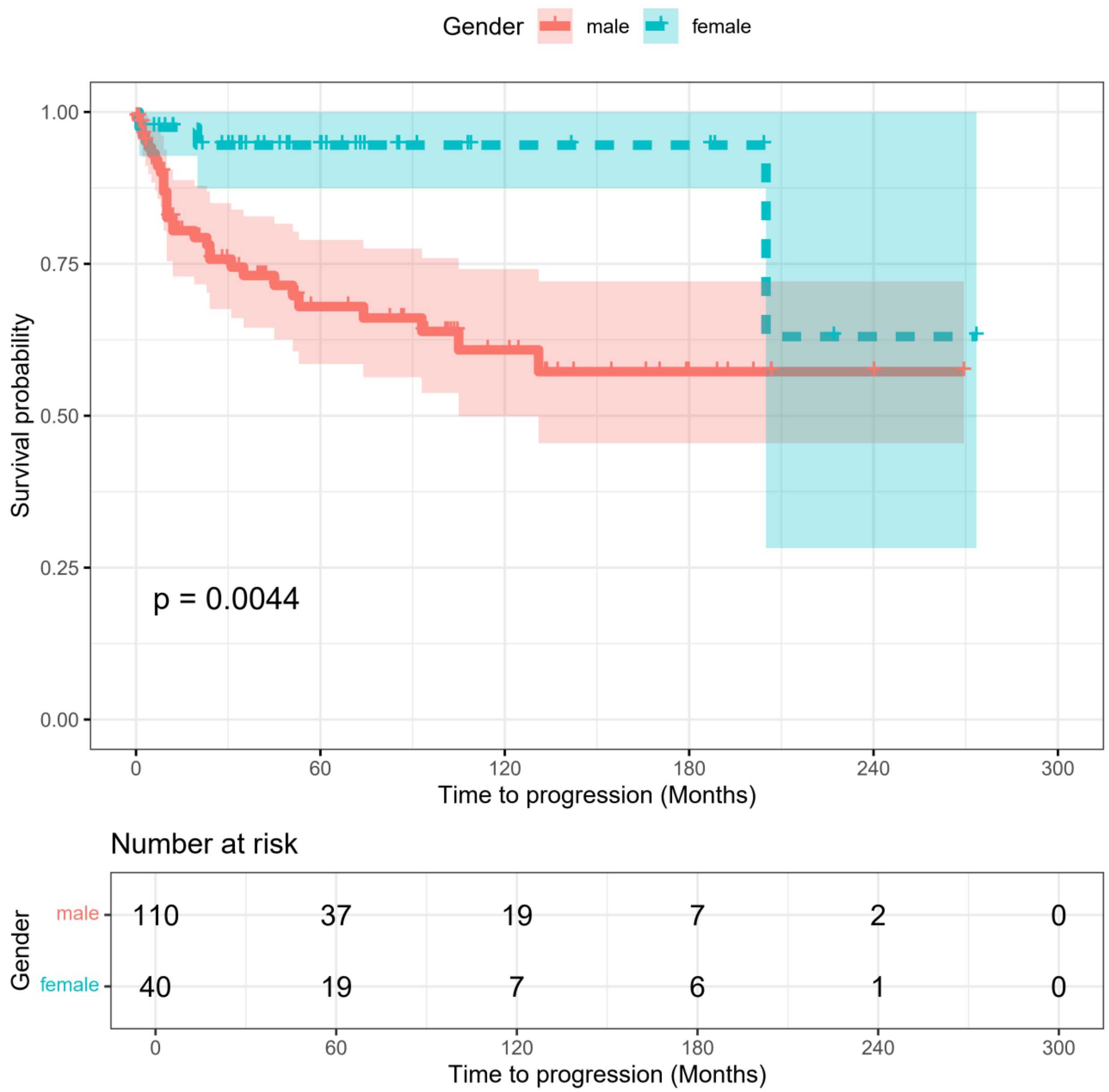

© 2025 Alsavaf MB et al. *JAMA Network Open*.

eFigure 11.

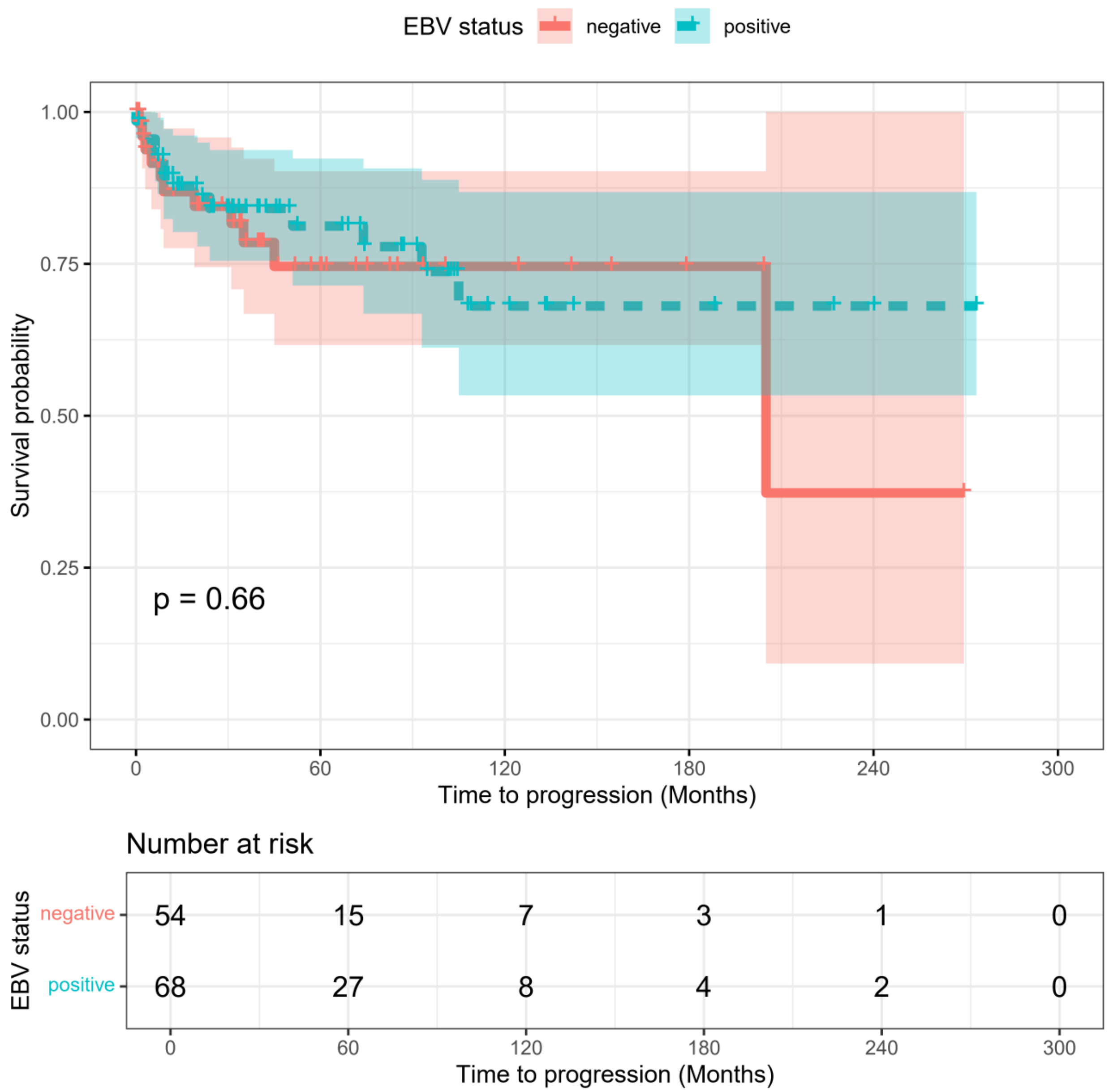

© 2025 Alsavaf MB et al. *JAMA Network Open*.

eFigure 12.

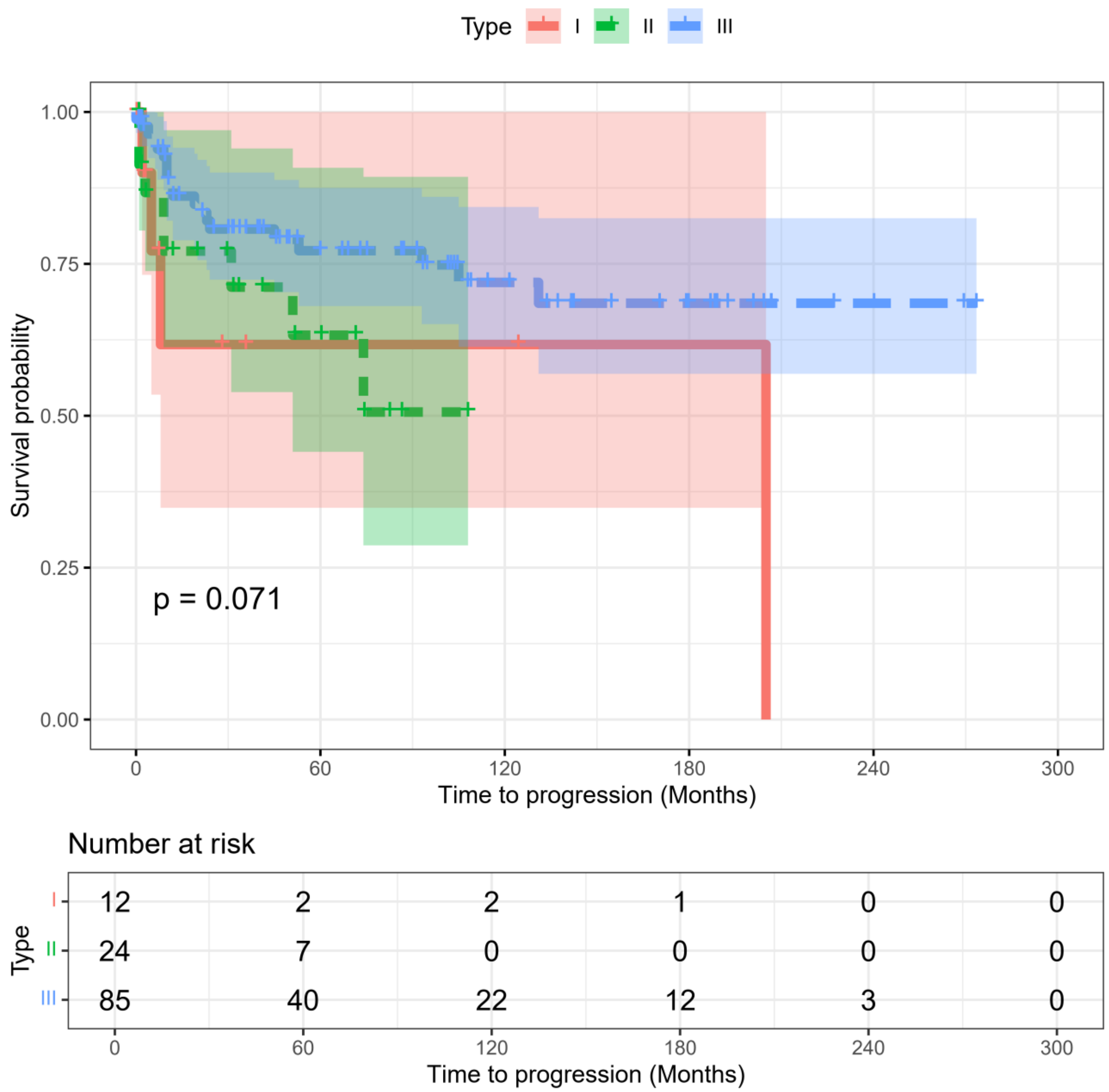

© 2025 Alsavaf MB et al. *JAMA Network Open*.

eFigure 13.

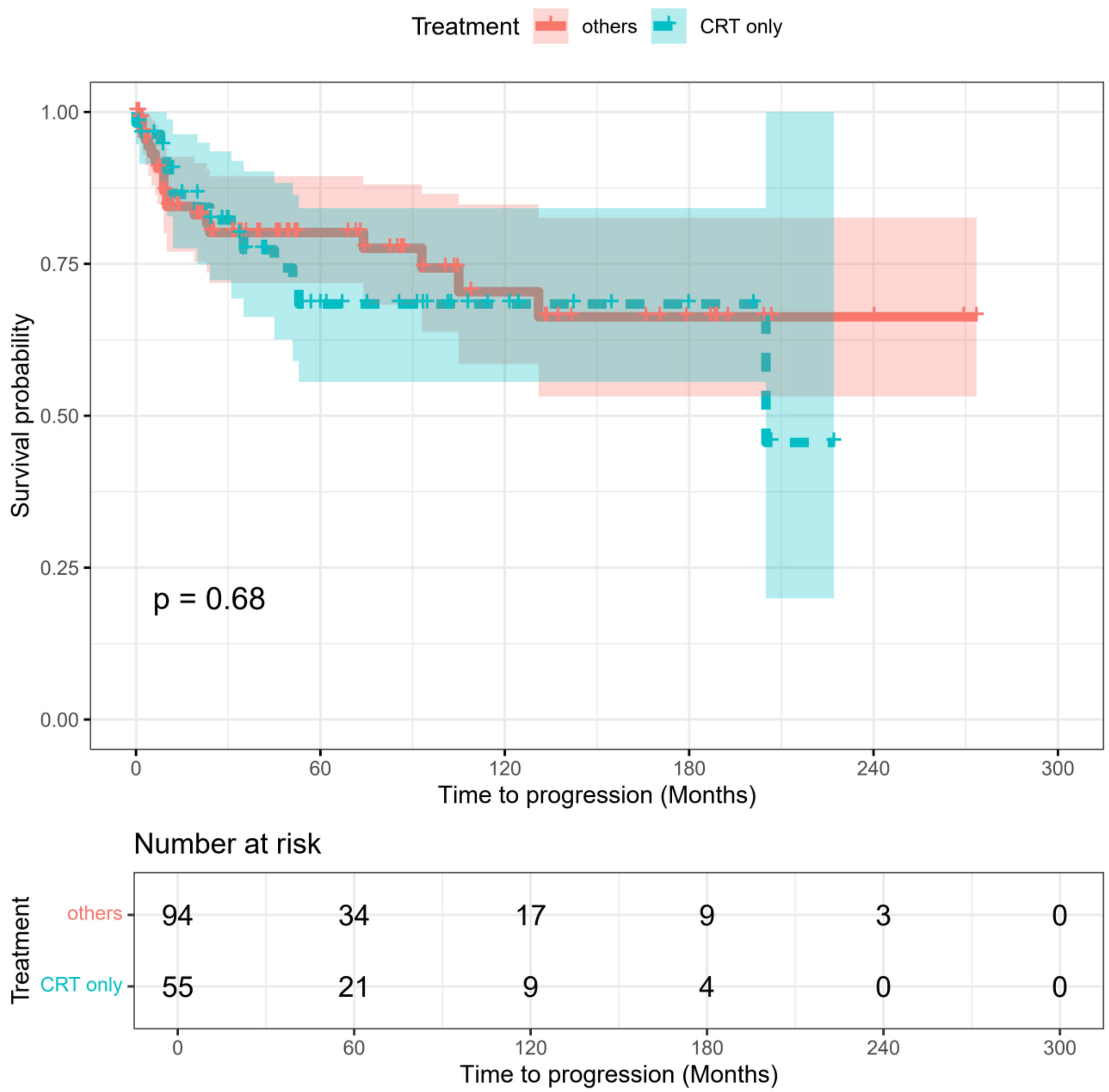

© 2025 Alsavaf MB et al. *JAMA Network Open*.

eTable 1. Univariate Cox regression analysis of potential prognostic factors

Variables		Recurrence		Metastasis		PFS		OS	
		Hazard Ratio (95% CI)	P-value	Hazard Ratio (95% CI)	P-value	Hazard Ratio (95% CI)	P-value	Hazard Ratio (95% CI)	P-value
Age at diagnosis		1.016 (0.986 – 1.046)	0.30	1.012 (0.985 – 1.039)	0.4	1.015 (0.992 – 1.039)	0.21	1.044 (1.024 – 1.064)	0.000
Sex	Male	Reference		Reference		Reference		Reference	
	Female	0.218 (0.051 – 0.935)	0.04	0.097 (0.013 – 0.716)	0.022	0.211 (0.065 – 0.689)	0.01	0.474 (0.248 – 0.906)	0.024
Race	White	Reference	0.58	Reference	0.10	Reference	0.33	Reference	0.26
	African	1.678 (0.625 – 4.5)	0.30	2.096 (0.905 – 4.851)	0.84	1.701 (0.789 – 3.664)	0.17	0.924 (0.488 – 1.748)	0.81
	Asian	1.085 (0.347 – 3.397)	0.89	0.519 (0.119 – 2.271)	0.38	0.869 (0.324 – 2.330)	0.78	0.515 (0.232 – 1.141)	0.10
Smoking	Current	Reference	0.084	Reference	0.61	Reference	0.45	Reference	0.001
	Former	2.497 (0.946 – 6.593)	0.06	1.451 (0.526 – 4.005)	0.47	1.717 (0.742 – 3.971	0.21	3.648 (1.797 – 7.406)	0.000
	Never	0.911 (0.289 – 2.874)	0.87	1.567 (0.630 – 3.899)	0.33	1.383 (0.619 – 3.090)	0.43	3.723 (1.778 – 7.793)	0.000
Alcohol	No	Reference		Reference		Reference		Reference	
	Yes	0.938 (0.391 – 2.249)	0.89	0.573 (0.241 – 1.365)	0.21	0.762 (0.372 – 1.561)	0.46	0.669 (0.393 – 1.141)	0.14
Marijuana	No	Reference		Reference		Reference		Reference	
	Yes	0.650 (0.151 – 2.794)	0.56	0.866 (0.259 – 2.901)	0.82	0.616 (0.188 – 2.021)	0.42	0.998 (0.491 – 2.026)	0.99
Histological type (WHO)	I	Reference	0.016	Reference	0.44	Reference	0.08	Reference	0.000
	II	0.6 (0.164 – 2.199)	0.44	1.048 (0.209 – 5.246)	0.95	0.770 (0.228 – 2.608)	0.67	0.409 (0.177 – 0.941)	0.036
	III	0.217 (0.068 – 0.692)	0.01	0.588 (0.134 – 2.572)	0.48	0.374 (0.127 – 1.106)	0.07	0.228 (0.114 – 0.455)	0.000
	I & II	Reference	0.36	Reference	0.10	Reference	0.19	Reference	0.000

© 2025 Alsavaf MB et al. *JAMA Network Open*.

**Stage (8th edition)**	**III**	1.799 (0.382 – 8.480)	0.46	4.597 (0.588 – 35.938)	0.15	2.008 (0.572 – 1.053)	0.28	1.141 (0.441 – 2.949)	0.79
	**IVa & IVb**	2.754 (0.603 – 12.582)	0.19	7.464 (0.985 – 56.541)	0.05	2.949 (0.863 – 10.069)	0.08	3.322 (1.409 – 7.833)	0.006
**EBV**	**Negative**	Reference		Reference		Reference		Reference	
	**Positive**	0.729 (0.281 – 1.894)	0.52	0.743 (0.286 – 1.930)	0.54	0.837 (0.379 – 1.846)	0.70	0.406 (0.222 – 0.745)	0.004
**P16**	**Negative**	Reference		Reference		Reference		Reference	
	**Positive**	0.624 (0.191 – 2.035)	0.43	1.468 (0.514 – 4.191)	0.47	0.759 (0.302 – 1.909)	0.56	1.784 (0.886 – 3.592)	0.10
**Treatment**	**CCRT**	Reference		Reference		Reference		Reference	
	**IC + CCRT / CCRT + AR**	1.038 (0.444 – 2.430)	0.93	1.005 (0.456 – 2.219)	0.99	1.152 (0.589 – 2.252)	0.68	1.189 (0.733 – 1.928)	0.48

© 2025 Alsavaf MB et al. *JAMA Network Open*.

eTable 2. Overall Survival (OS) Rates

Category		Median OS (months)	12-month OS	36-month OS	60-month OS	120-month OS	p-value
**Overall**		104	80.4%	70.2%	60.9%	47.9%	-
**Sex**	**Female**	204	85.6%	83%	75.9%	63.3%	0.027
	**Male**	86	78.5%	65.7%	55.7%	42.6%	
**Race**	**African American**	124	87.50%	74.54%	58.38%	51.89%	0.19
	**Asian**	192	100%	84.21%	78.20%	62.56%	
	**White**	93	74.39%	66.14%	57.83%	44.36%	
**Smoking Status**	**Never**	-	92.6%	85.7%	79.4%	71.3%	<.001
	**Former**	-	70.2%	58%	47.3%	41.4%	
	**Current**	-	77%	65.8%	55.1%	34%	
**EBV Status**	**Negative**	-	68.9%	63.3%	50.5%	46.3%	<.001
	**Positive**	-	89.7%	84.4%	79.5%	60.8%	
**WHO Grade**	**I**	-	26.7%	26.7%	26.7%	26.7%	<.0001
	**II**	-	76%	66.7%	55%	-	
	**III**	-	89.7%	77.0%	67.9%	56.6%	
**Stage**	**I & II**	-	88.57%	83.36%	76.94%	76.94%	<.0001
	**III**	-	94.34%	88.00%	78.92%	57.92%	
	**IVa &IVb**	-	68.40%	52.79%	41.69%	28.6%	

© 2025 Alsavaf MB et al. *JAMA Network Open*.

eTable 3. Progression-Free Survival (PFS) Rates

Category		Median OS (months)	12-month OS	36-month OS	60-month OS	120-month OS	p-value
**Overall**		-	86%	79.7%	74.6%	68.7%	-
**Sex**	**Female**	-	100%	97.1%	92.2%	92.2%	0.004
	**Male**	-	80.4%	72.9%	67.7%	60.5%	
**EBV Status**	**Negative**	-	88.84%	80.28%	71.78%	71.78%	<.001
	**Positive**	-	87.87%	84.17%	81.27%	68.11%	
**WHO Grade**	**I**	-	61.71%	61.71%	61.71%	61.71%	0.07
	**II**	-	81.51%	75.69%	59.6%	-	
	**III**	-	86.07%	80.53%	76.81%	71.36%	
**Stage**	**I & II**	75	96%	90.9%	82.7%	82.7%	0.14
	**III**	46	92.1%	80.1%	76.9%	62.3%	
	**IVa &IVb**	42	75.3%	73%	65.7%	65.7%	

© 2025 Alsavaf MB et al. *JAMA Network Open*.

© 2025 Alsavaf MB et al. *JAMA Network Open*.
