# Supplementary material for: Patient Characteristics and Treatment Outcomes of Nasopharyngeal Carcinoma in Nonendemic Regions
Source: JAMA Netw Open. 2025 Mar 26;8(3):e251895. doi: 10.1001/jamanetworkopen.2025.1895 (PMC11947841; doi:10.1001/jamanetworkopen.2025.1895)
Supplement: Supplement 2. — Data Sharing Statement [file jamanetwopen-e251895-s002.pdf]

Data Sharing Statement

Alsavaf. Patient Characteristics and Treatment Outcomes of Nasopharyngeal Carcinoma in Nonendemic Regions. *JAMA Netw Open*. Published March 26, 2025.
doi:10.1001/jamanetworkopen.2025.1895

Data
Data available: No
